# TRENDS IN HOSPITALIZATIONS, COSTS, AND IN-HOSPITAL MORTALITY IN PRIMARY BILIARY CHOLANGITIS: A DECADE OF REAL-WORLD EVIDENCE FROM BRAZIL’S UNIFIED HEALTH SYSTEM

**DOI:** 10.1590/S0004-2803.24612026-070

**Published:** 2026-10-30

**Authors:** Guilherme Grossi Lopes CANÇADO, Cláudia Alves COUTO, Luciana Costa FARIA, Bernardo de Faria MORAES, Paulo Lisboa BITTENCOURT, Cristiane Alves Villela-Nogueira, Vivian ROTMAN, Debora Raquel Benedita TERRABUIO, Eduardo Luiz Rachid CANÇADO, Maria Lúcia FERRAZ

**Affiliations:** 1 Universidade Federal de Minas Gerais, Hospital das Clínicas, Instituto Alfa de Gastroenterologia, Belo Horizonte, MG, Brasil.; 2 Hospital da Polícia Militar de Minas Gerais, Departamento de Gastroenterologia e Hepatologia, Belo Horizonte, MG, Brasil.; 3 Hospital Português, Salvador, BA, Brasil.; 4 Universidade Federal do Rio de Janeiro, Faculdade de Medicina, Hospital Universitário Clementino Fraga Filho, Departamento de Clínica Médica, Rio de Janeiro, RJ, Brasil.; 5 Universidade de São Paulo, Departamento de Gastroenterologia, São Paulo, SP, Brasil.; 6 Universidade Federal de São Paulo, Disciplina de Gastroenterologia, São Paulo, SP, Brasil.

**Keywords:** Primary biliary cirrhosis, mortality, hospitalization, epidemiology, Latin America, Brazil, Cirrose biliar primária, mortalidade, hospitalização, epidemiologia, América Latina, Brasil

## Abstract

**Background:**

Primary biliary cholangitis (PBC) is a rare autoimmune liver disease with limited epidemiological data from Latin America.

**Objectives:**

To analyzed nationwide trends in PBC-related hospitalizations and in-hospital mortality within Brazil’s Unified Health System (SUS) from 2015 to 2024.

**Methods:**

National hospital admission data for ICD-10 code K74.3 were analyzed. Hospitalization rates per 100,000 inhabitants, in-hospital mortality, costs, and mean length of stay were estimated. Temporal changes were assessed using interrupted time-series segmented regression, and descriptive analyses were stratified into pre-pandemic, pandemic (COVID-19), and post-pandemic periods.

**Results:**

Between 2015 and 2024, 2,913 hospitalizations were recorded. The annual hospitalization rate ranged from 0.11 per 100,000 in 2022 to 0.19 per 100,000 in 2020, with a mean of 0.15 per 100,000 over the study period. Mean admission-level in-hospital mortality was 15.4%, ranging from 11.7%-18.5%. Mean length of stay was 7.9 days, with little variation over time. A transient increase in hospitalizations was observed in 2020 (β_2_ = +0.046, *P*=0.03), followed by a negative slope change in the post-2020 period (β_2_ = −0.009, *P*=0.04), suggesting temporary disruption related to the COVID-19 pandemic. The Southeast had the highest hospitalization burden. Inflation-adjusted expenditures totaled R$ 11.38 million (USD 2,112,227.43), with a mean cost of R$ 3,908 (USD 725.10) per admission.

**Conclusion:**

PBC-coded hospitalizations in Brazil are infrequent but associated with substantial in-hospital mortality. Strengthening outpatient management and regional equity in hepatology care remains a public health priority.

## HIGHLIGHTS

• This nationwide large real-world analysis describes trends in hospitalizations for Primary Biliary Cholangitis (PBC) within Brazil’s Unified Health System (SUS) from 2015 to 2024.

• Hospitalization rates remained low (mean 0.15 per 100,000) but stable, with a transient surge during the COVID-19 pandemic followed by a decline.

• In-hospital mortality averaged 15%, substantially higher than in high-income countries, while the mean hospital stay remained consistent at eight days.

• Inflation-adjusted expenditures totaled R$ 11.38 million (USD 2,112,227.43) over the period, with substantial regional variation and a mean cost of R$ 3,908 (USD 725.10) per admission.

• These findings suggest a persistent burden of advanced or clinically complex PBC requiring inpatient care and indicate potential regional differences in access, coding, referral pathways, and specialized hepatology services.

## RESUMO

**Contexto:**

A colangite biliar primária (CBP) é uma doença hepática autoimune rara, com dados epidemiológicos escassos na América Latina.

**Objetivo:**

Analisar as tendências nacionais de hospitalizações relacionadas à CBP e de mortalidade intra-hospitalar no Sistema Único de Saúde (SUS) do Brasil entre 2015 e 2024.

**Métodos:**

Foram analisados dados nacionais de internações hospitalares referentes ao código CID-10 K74.3. Foram estimadas as taxas de hospitalização por 100.000 habitantes, mortalidade intra-hospitalar, custos e tempo médio de permanência hospitalar. As mudanças temporais foram avaliadas por meio de regressão segmentada de séries temporais interrompidas, e as análises descritivas foram estratificadas nos períodos pré-pandêmico, pandêmico (COVID-19) e pós-pandêmico.

**Resultados:**

Entre 2015 e 2024, foram registradas 2.913 hospitalizações. A taxa anual de hospitalização variou de 0,11 por 100.000 habitantes em 2022 a 0,19 por 100.000 habitantes em 2020, com média de 0,15 por 100.000 no período do estudo. A mortalidade intra-hospitalar média por internação foi de 15,4%, variando de 11,7% a 18,5%. O tempo médio de permanência hospitalar foi de 7,9 dias, com pouca variação ao longo do tempo. Observou-se um aumento transitório das hospitalizações em 2020 (β_2_ = +0,046; *P*=0,03), seguido por uma mudança negativa na inclinação no período pós-2020 (β_3_ = −0,009; *P*=0,04), sugerindo uma interrupção temporária relacionada à pandemia de COVID-19. A região Sudeste apresentou a maior carga de hospitalizações. Os gastos ajustados pela inflação totalizaram R$ 11,38 milhões (USD 2.112.227,43), com custo médio de R$ 3.908 (USD 725,10) por internação.

**Conclusão:**

As hospitalizações relacionadas à CBP no Brasil são infrequentes, mas associadas a elevada mortalidade intra-hospitalar. A melhoria do manejo ambulatorial e da equidade regional no acesso ao cuidado hepatológico permanece uma prioridade de saúde pública.

## INTRODUCTION

Primary biliary cholangitis (PBC) is a chronic, immune-mediated cholestatic liver disease characterized by progressive destruction of intrahepatic bile ducts, leading to biliary cirrhosis and, ultimately, end-stage liver failure^1^ ^,^ ^2^. Although historically considered rare, the global prevalence of PBC has increased over recent decades^3^, partly due to improved diagnostic awareness, broader availability of serological testing for anti-mitochondrial antibodies (AMA), and enhanced survival with ursodeoxycholic acid (UDCA) therapy and second-line therapies^4^. Despite therapeutic advances, PBC continues to impose significant morbidity, with a subset of patients developing portal hypertension, hepatic decompensation, or hepatocellular carcinoma^5^ ^,^ ^6^.

Epidemiological studies have provided valuable insights into the natural history and healthcare burden of PBC. A recent global meta-analysis estimated a pooled prevalence of approximately 18 cases per 100,000 individuals and an incidence of 1.8 per 100,000 person-years^3^. Contemporary data from Europe^7^ ^,^ ^8^, North America^9^, and the Asia-Pacific region^10^ ^-^ ^13^ consistently demonstrated a marked upward trend in prevalence over recent decades. In North America, population-based analyses have reported annual hospitalization rates ranging from 1.7 to 3.7 per 100,000 inhabitants, with evidence of a progressive increase over time^14^ ^,^ ^15^. Mortality related to advanced PBC has decreased^15^, largely reflecting timely diagnosis and effective first-line and second-line therapies. However, data from low- and middle-income countries, particularly from Latin America, where most of the new second-line drugs are still not available, remain extremely scarce.

In Brazil, the world’s fifth-largest country, the Public Unified Health System (Sistema Único de Saúde - SUS) provides comprehensive healthcare access to over 210 million inhabitants. The SUS is one of the largest publicly funded health systems globally, covering most of the hospitalizations, including those for chronic liver diseases. Administrative hospital databases within the SUS have been increasingly used for epidemiological monitoring of different conditions. Indeed, real-world data (RWD) analyses provide a pragmatic means to evaluate population-level trends in rare diseases like PBC, and are particularly relevant in resource-constrained settings, where disease registries are limited.

The present study addresses this evidence gap by providing a comprehensive, decade-long analysis of hospitalizations due to PBC in Brazil’s Unified Health System. We used official national administrative data from 2015 through 2024 to describe trends in hospitalization rates, in-hospital mortality, and length of stay, while evaluating the potential impact of the COVID-19 pandemic through segmented time-series modeling.

## METHODS

### Database

This was a nationwide, ecological, time-series analysis of hospitalizations for PBC (ICD-10 K74.3) in Brazil between January 1, 2015, and December 31, 2024. The study used data from the Techtrials Epiwriting Knowledge Base®, which integrates official administrative records from the Brazilian Unified Health System (SUS) and population estimates from the Brazilian Institute of Geography and Statistics (IBGE), using artificial intelligence technology (ETLs and Webcrawlers). 

### Statistical analysis

All analyses were conducted using anonymized, population-level administrative data from the Brazilian Unified Health System (SUS). No patient-level data were accessed. Hospitalization events for Primary Biliary Cholangitis were identified by the ICD-10 code K74.3 at hospital admission, aggregated by year and geographic macroregions [North, Northeast, Southeast, South, and Central-West]. Descriptive analyses were first performed to summarize hospitalization counts, mean costs per hospitalization, in-hospital deaths, and average length of stay. Annual and regional indicators were calculated to provide national and subnational perspectives across the study period (2015-2024).

Hospitalization rates were expressed as the number of PBC-coded admissions per 100,000 inhabitants. For the primary descriptive analysis, state-level IBGE 2025 population projections were used as a fixed reference denominator. This strategy was chosen to generate internally comparable standardized hospitalization indicators across years and regions, minimizing variation in rates caused solely by annual demographic fluctuations, intercensal projection updates, or changes in the underlying reference population. Therefore, these rates should be interpreted as standardized admission-level hospitalization indicators rather than year-specific incidence rates or patient-level disease frequency estimates. Because Brazil’s total population changed modestly during the study period, from approximately 204.5 million in 2015 to 213.4 million in 2025, the use of a fixed denominator is unlikely to change the overall direction of national temporal patterns, although it may slightly affect absolute annual estimates and regional rates in areas with more dynamic demographic changes. In-hospital mortality was defined as the ratio of deaths to total hospitalizations and expressed as a percentage. Mean length of stay was calculated as the weighted average of individual hospitalizations multiplied by their respective lengths of stay, divided by the total number of hospitalizations. The mean cost per hospitalization in the public system was computed by dividing the total cost by the number of hospitalizations in each stratum. All nominal costs were converted to constant 2024 BRL using IPCA (Extended National Consumer Price Index) annual inflation rates (retrieved from official IBGE-based historical series) as a proxy index for medical inflation. Because all monetary values in the analysis had already been standardized to 2024 BRL through inflation adjustment using the Brazilian IPCA index, cost conversion to USD was performed using a single exchange rate for the base year. For this purpose, we applied the average 2024 BRL-USD exchange rate of 5.39 BRL per USD (mean rate provided by the Brazilian Central Bank for 2024), which was uniformly used across all inflation-adjusted cost components. As administrative records may include multiple admissions for the same patient, “number of hospitalizations” was not interpreted as distinct individuals. Therefore, repeated admissions by the same person could not be identified or linked across time. All hospitalization rates, mortality proportions, length-of-stay estimates, and cost estimates should be interpreted as admission-level indicators. The terms “hospitalizations” and “admissions” are used throughout the manuscript to refer to events rather than unique patients.

Temporal patterns were examined using descriptive trend analysis and stratified into three epidemiologically relevant periods: pre-pandemic (2015-2019), pandemic (2020-2021), and post-pandemic (2022-2024). This segmentation was selected to explore potential structural breaks in healthcare delivery related to the COVID-19 pandemic and its aftermath. To formally assess changes in hospitalization rates over time, an Interrupted Time Series (ITS) analysis was conducted at the national level. The ITS model evaluated both immediate (level) and gradual (trend) changes in the annual hospitalization rate following the onset of the pandemic in 2020. The model specification was: *Y*_*t*_ = β_0_ + β_1_t + β_2_D_2020_ + β_3_t_post2020_ + ε_t_, where *Y*_*t*_ represents the annual hospitalization rate per 100,000 inhabitants, *t* is a continuous variable indicating time in years since 2015, D_2020_ is a binary indicator for the post-2020 period, and t_post2020_ represents the number of years after 2020. The coefficients β_2_ and β_3_ estimate the immediate level change and change in slope following the pandemic onset, respectively. Parameters were estimated using Ordinary Least Squares (OLS) regression. Approximate p-values were calculated under the assumption of normality of the residuals, and statistical significance was set at *P*<0.05 (two-sided). Because the ITS analysis was based on only 10 annual observations, it was considered exploratory and hypothesis-generating. The small number of time points limits statistical power, precision of coefficient estimates, formal assessment of autocorrelation, and robustness of residual diagnostics. Accordingly, ITS coefficients were used to summarize temporal patterns rather than to establish a causal effect of the COVID-19 pandemic.

All analyses and figure generation were performed in Python 3.11 using the pandas (v2.2) and matplotlib (v3.9) libraries within the Epiwriting analytical environment (version 2025.10). Results are reported in accordance with NICE and EMA real-world evidence (RWE) methodological standards for ecological time-series studies. Ninety-five percent confidence intervals were calculated for the main descriptive estimates whenever appropriate. Because length of stay and cost data were available only as aggregated annual means without admission-level variance estimates, confidence intervals were not calculated for these continuous indicators.

## RESULTS

Between 2015 and 2024, a total of 2,913 hospitalizations for PBC were recorded in the Brazilian Unified Health System (SUS), with 448 in-hospital deaths (Table 1). These admissions occurred across all 27 federative units, ensuring nationwide representativeness. Each record represented a hospitalization event rather than an individual patient, in accordance with the methodological criteria of the study.

**TABLE 1:** Summary of indicators for PBC hospitalizations in Brazil (SUS), 2015-2024. National annual figures for hospitalization rate (per 100,000 inhabitants), in-hospital mortality (%), mean length of stay (days) and inflation-adjusted mean cost per hospitalization in BRL (USD).

Year	Hospitalization (n)	Deaths (n)	Hospitalization rate (/100,000) (95%CI)	In-Hospital Mortality (%) (95%CI)	Mean length of stay (days)	Mean Cost per Hospitalization in BRL (USD)
2015	297	41	0.139 (0.123-0.155)	13.8 (10.3-18.2)	8.3	2660.19 (493.50)
2016	257	41	0.120 (0.106-0.135)	16.0 (12.0-20.9)	8.7	4978.69 (923.69)
2017	262	35	0.123 (0.108-0.138)	13.4 (9.8-18.0)	9.1	4003.50 (742.76)
2018	277	41	0.130 (0.115-0.145)	14.8 (11.1-19.5)	8.7	4391.31 (814.72)
2019	308	57	0.144 (0.128-0.160)	18.5 (14.6-23.2)	7.7	4239.13 (786.48)
2020	414	63	0.194 (0.175-0.213)	15.2 (12.1-19.0)	6.1	3392.44 (629.40)
2021	362	64	0.170 (0.152-0.187)	17.7 (14.1-21.9)	7.0	2870.25 (532.51)
2022	242	42	0.113 (0.099-0.128)	17.4 (13.1-22.6)	7.7	4712.96 (874.39)
2023	248	29	0.116 (0.102-0.131)	11.7 (8.3-16.3)	8.8	4332.56 (803.81)
2024	246	35	0.115 (0.101-0.130)	14.2 (10.4-19.1)	7.5	4413.94 (818.91)

### Hospitalization rates and COVID-19 pandemic impact

At the national level, the annual hospitalization rate ranged from 0.11 per 100,000 inhabitants in 2022 to 0.19 per 100,000 in 2020, with an overall mean of 0.15 per 100,000 during the ten-year period (Figure 1). A moderate upward trend was observed until 2020, followed by a decline in the subsequent years. The in-hospital mortality rate averaged 15.4%, fluctuating between 11.7% (2023) and 18.5% (2019), without a consistent long-term trend (Figure 2).

**FIGURE 1:**
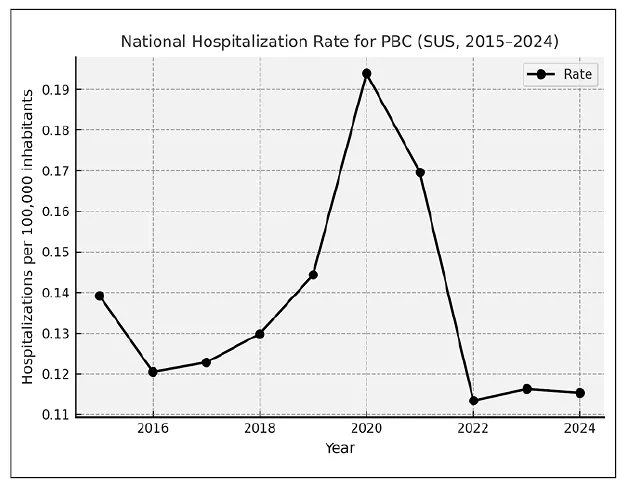
Temporal trend in hospitalization rates for Primary Biliary Cholangitis (PBC; ICD-10 K74.3) in the Brazilian Unified Health System (SUS), 2015-2024. Annual hospitalization rates per 100,000 inhabitants, standardized by the 2025 IBGE population. A transient increase in admissions is observed during 2020-2021, followed by a gradual decline.

**FIGURE 2:**
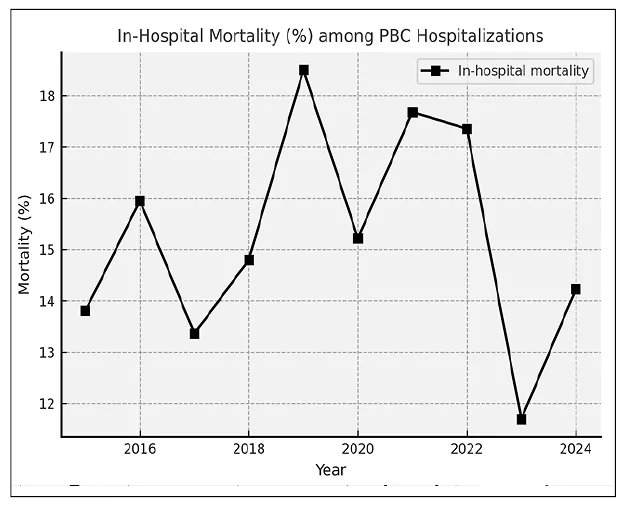
In-hospital mortality rate (%) among PBC-related hospitalizations in SUS, 2015-2024. National annual in-hospital mortality proportions, showing rates of approximately 15% throughout the decade, with only modest year-to-year variation.

When stratified by region, the Southeast exhibited the highest mean hospitalization rate (0.21 per 100,000), followed by the South (0.18) and Northeast (0.13). The North and Central-West regions consistently showed lower rates (both below 0.10 per 100,000). These regional differences persisted across the studied periods. However, since this analysis was based on administrative admission records, it is not possible to establish whether this reflects differences in disease prevalence, disease severity, access to care or quality of data management. Compared with the pre-pandemic baseline (2015-2019), mean hospitalization rates increased during the pandemic period (2020-2021) and subsequently decreased during the post-pandemic period (2022-2024), a pattern observed in all major regions of the country. Specifically, between the pre- and post-pandemic phases, hospitalization rates declined by approximately 10% in the Southeast, 9% in the Central-West, 8% in the Northeast, and 6% in the South. The North region demonstrated the largest relative decline (12%) and maintained the lowest rates throughout. These descriptive patterns indicate geographic heterogeneity in PBC-coded hospitalization rates across Brazilian macroregions, with lower recorded rates in the North and Central-West regions throughout the study period (Table 2).

The exploratory Interrupted Time Series (ITS) analysis supported the descriptive findings. The segmented regression model revealed a statistically significant level increase in 2020 (β_2_ = +0.046, *P*=0.03) and a negative slope change in the post-pandemic period (β_3_ = −0.009, *P*=0.04). This indicates an immediate, short-term rise in PBC hospitalizations during the early pandemic period, followed by a gradual decline toward pre-pandemic levels. The pre-pandemic time trend (β_1_ = +0.008, *P*=0.02) was positive, reflecting the slow but steady increase observed between 2015 and 2019 (Figure 3, Table 3). However, because the model was based on only 10 annual observations, these estimates should be interpreted cautiously as a descriptive summary of temporal variation coinciding with the COVID-19 period rather than as evidence of a definitive causal pandemic effect.

**FIGURE 3:**
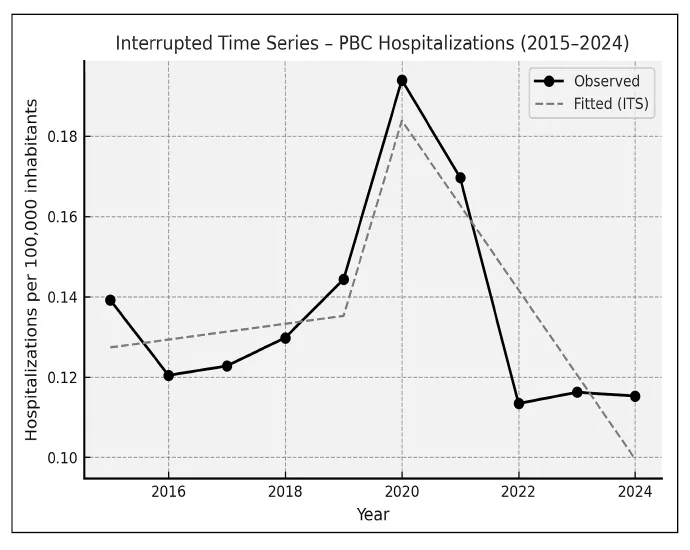
Interrupted Time Series (ITS) analysis of national PBC hospitalization rates (2015-2024). Observed versus fitted values from segmented regression. A significant level increase was observed in 2020 (β_2_ = +0.046, p = 0.03), followed by a negative slope change in the post-2020 period (β_3_ = −0.009, p = 0.04), suggesting a transient disruption related to the COVID-19 pandemic.

**TABLE 2:** Hospitalization rates for PBC per region and epidemiological period. Aggregated rates (per 100,000 inhabitants) across the Pre-pandemic (2015-2019), Pandemic (2020-2021), and Post-pandemic (2022-2024) periods, including relative change (Δ) compared to baseline.

Region	Pre (2015-2019)	Pandemic (2020-2021)	Post (2022-2024)	Δ (Post vs. Pre)
North	0.08	0.10	0.07	−12%
Northeast	0.12	0.15	0.11	−8%
Southeast	0.19	0.23	0.17	−10%
South	0.17	0.20	0.16	−6%
Central-West	0.11	0.14	0.10	−9%

(Rates per 100,000 inhabitants, standardized to 2025 population).

**TABLE 3:** Interrupted Time Series (ITS) model coefficients for national hospitalization rate (2015-2024).

Parameter	Estimate (β)	Std. Error	t	***P* (approx.)**	95%CI
Intercept (β_0_)	0.098	0.012	8.2	<0.001	0.069-0.127
Pre-2020 trend (β_1_)	+0.008	0.003	2.6	0.024	0.001-0.015
Level change 2020 (β_2_)	+0.046	0.020	2.3	0.031	-0.003-0.095
Post-2020 trend change (β_3_)	−0.009	0.004	−2.4	0.041	-0.019-0.001

Parameter estimates (β), standard errors, t-statistics, and p-values from the segmented OLS regression model.

The ITS model demonstrated a statistically significant level increase in 2020 followed by a negative post-pandemic trend.

### In-hospital mortality and length of stay

The in-hospital mortality rate averaged 15.4%, ranging from 11.7% in 2023 to 18.5% in 2019. Mortality was relatively stable over time, with a slight increase during the pandemic years (2020-2021), followed by a gradual reduction thereafter. Despite these fluctuations, no sustained downward trend was observed throughout the decade. Mortality remained consistently high across all years. When analyzed by region, in-hospital mortality was highest in the North (16.9%) and Northeast (16.3%), intermediate in the Southeast (15.2%) and South (14.8%), and lowest in the Central-West (13.9%).

The average length of hospital stay (LOS) was 7.9 days overall, with limited variation across the period studied. The shortest mean LOS occurred in 2020 (6.1 days) and the longest in 2017 (9.1 days) (Figure 4). The length of stay remained relatively uniform across geographic regions, ranging from 7.6 days in the South to 8.2 days in the Northeast. 

**FIGURE 4:**
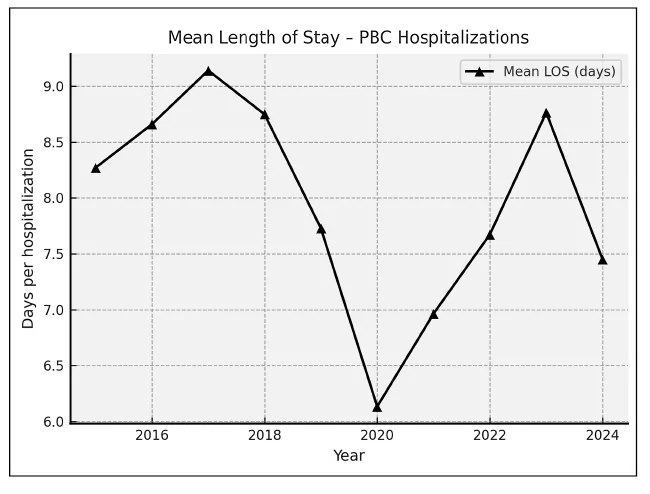
Mean length of stay (days) for PBC hospitalizations in SUS, 2015-2024. The mean duration of hospitalization remained relatively stable over time, averaging approximately 8 days, suggesting broadly consistent inpatient management despite pandemic-related pressures.

### Annual costs related to hospitalizations

Overall, hospitalizations for PBC generated a total cost of R$ 11.38 million (USD 2,112,227.43) in the Brazilian public health system over 2015-2024, with a mean inflation-adjusted cost of R$ 3,908 (USD 725.10) per admission. Annual costs increased from approximately R$ 0.79 million in 2015 to a peak of R$ 1.40 million in 2020, remaining around R$ 1.0-1.3 million in the subsequent years. The mean cost per hospitalization was lowest in 2015 (R$ 2,660) and highest in 2016 (R$ 4,979), with most years falling between R$ 3,400 and R$ 4,700. The Southeast region presented the highest mean cost per hospitalization [R$ 5,720 compared to South (R$ 2,835), Central West (R$ 2,458), Northeast (R$ 2,151) and North (R$ 1,480)], with an inflation-adjusted total cost of approximately R$ 7.57 million (2015-2024), representing the majority of national spending. A graphical summary is presented in Figure 5.

**FIGURE 5:**
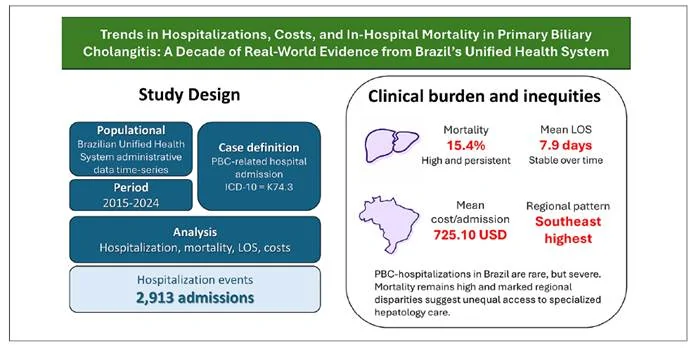
Graphical summary of the study findings.

## DISCUSSION

This nationwide, population-based study provides the first comprehensive real-world assessment of hospitalizations for PBC within the Brazilian public Unified Health System (SUS) and, to our knowledge, the first data of this kind from Latin America. Covering the period from 2015 to 2024, this analysis shows that PBC remains a rare but clinically significant cause of hospitalization in Brazil, characterized by stable hospitalization rates, persistently high in-hospital mortality, and a relatively consistent length of stay.

The mean annual hospitalization rate for PBC in Brazil was 0.15 per 100,000 inhabitants, markedly lower than the 1.7-3.7 per 100,000 reported in North America^14^ ^,^ ^15^. This disparity likely reflects differences in diagnostic awareness, healthcare access, and registry ascertainment rather than true variations in disease burden. In the United States, a nationwide inpatient study^14^ revealed notable racial disparities, with hospitalization rates 12% higher among Hispanics, 53% lower among Blacks, and 5% lower among other racial minorities compared with Whites. These discrepancies reflect how variations in PBC prevalence, early-onset or aggressive disease, structural healthcare inequities, and limited UDCA use or reduced response collectively shape hospitalization rates and outcomes across racial and ethnic groups^16^ ^-^ ^18^. It is important to mention that direct comparisons are limited by major methodological differences, including coding strategies, population denominators, healthcare system structure, admission thresholds, patient-level linkage, and case-mix adjustment. Therefore, these international data should be viewed as contextual benchmarks rather than directly equivalent estimates.

The scarcity of comparable epidemiological data from Latin America further underscores the relevance of the present study as a baseline reference for the region. The observed stability in hospitalization rates across most of the decade suggests that the burden of advanced PBC requiring inpatient care has remained constant, possibly indicating both the chronicity of disease progression and the plateau in access to effective outpatient therapies such as UDCA.

The admission-level in-hospital mortality proportion observed in this study was approximately 15%, which appears higher than estimates reported in large North American inpatient analyses, where in-hospital rates typically fall below 8%^14^ ^,^ ^15^ ^,^ ^19^. Moreover, a large North American inpatient analysis from 2005-2014 reported not only a low baseline mortality of 3.8%, but also a significant downward trend over the decade, with the rate falling to 2.8% by 2014^15^. A previous study demonstrated that, unlike many countries, Brazil saw a steady rise in liver-disease mortality from 1996 to 2021, representing ~3% of all deaths, with a mortality rate by liver disease of 19% in 2021^20^. However, this data should be interpreted cautiously because differences in coding practices, study methods, admission thresholds, healthcare access, case mix, and the ability to identify repeated admissions may substantially influence mortality estimates, making comparisons with international inpatient datasets extremely hard. Importantly, the present database does not provide cause-specific mortality, and deaths could not be classified as liver-related, PBC-attributable, procedure-related, infection-related, cardiovascular, malignant, or due to other causes. Therefore, the observed mortality should not be interpreted as PBC-specific mortality. Rather, it likely reflects the severity and complexity of hospitalization events coded as PBC within the public health system, potentially including advanced liver disease, comorbid conditions, delayed presentation, and non-liver-related causes of death. Indeed, we previously showed that approximately one-third of patients with PBC in Brazil are diagnosed at the cirrhotic stage^21^. Furthermore, limited access to liver transplantation centers, which are usually concentrated in major Brazilian state capitals, likely limits treatment options. Taken together, these elements suggest that mortality may reflect structural inequities rather than intrinsic differences in disease biology and likely reflects the clinical complexity and case mix of admissions coded as PBC within the public hospital system.

Regional variation in hospitalization rates was observed, with higher recorded rates in the Southeast and South and lower rates in the North and Central-West. These differences may reflect several non-mutually exclusive factors, including regional variation in diagnostic capacity, referral patterns, availability of specialized hepatology services, coding practices, hospital admission thresholds, and true differences in disease burden^22^ ^,^ ^23^. Because patient-level clinical data and outpatient diagnostic information were not available, this study cannot determine the relative contribution of these mechanisms. Therefore, regional findings should be interpreted as signals of heterogeneity in recorded inpatient care rather than direct evidence of causal disparities in PBC biology or management.

The ITS analysis provided an exploratory description of temporal changes in PBC-coded hospitalizations around the COVID-19 period. The observed increase in 2020 and subsequent decline may be compatible with changes in healthcare utilization, outpatient disruption, delayed presentation, or coding/admission practices during the pandemic period. However, the annual aggregation, limited number of time points, and absence of patient-level data preclude causal attribution to the pandemic, although a pandemic-related impact remains biologically and epidemiologically plausible. For instance, a large nationwide study conducted in Japan^24^ documented a significant 21% rise in the admission rate for alcohol-associated liver disease (ALD) and cirrhosis during the COVID-19 epidemic compared to the pre-epidemic period. This trend was also observed in the United States, in which a nationwide, population-based study^25^ documented increased hospitalizations for ALD during the COVID-19 pandemic. Furthermore, a single-centre retrospective study in the United Kingdom^26^ reported that, although overall admissions for decompensated cirrhosis remained stable during the COVID-19 pandemic, patients admitted in this period exhibited significantly higher Model for End-Stage Liver Disease (MELD) scores and experienced increased hospital readmission rates. In Brazil, the observed peak in 2020 may reflect a similar phenomenon: restricted ambulatory access, delayed diagnostic and laboratory services, and a backlog of advanced cases presenting acutely. The subsequent decline in hospitalization rates may reflect partial restoration of outpatient care continuity and broader healthcare system recovery.

The length of hospital stay, which averaged eight days throughout the study period, remained consistent with values reported internationally. In a nationwide United States analysis of more than 22,000 hospitalized cases with PBC^15^, a mean length of stay was reported as 6.9 days in 2005 and 5.4 days in 2014, indicating a downward trend consistent with improvements in inpatient management and care efficiency. Similarly, another study conducted in the United States^14^ reported a relatively stable mean annual length of stay of approximately 6.8 days per hospitalization (ranging from 7.5 in 2007 to 6.7 in 2014), reinforcing the overall consistency of inpatient care patterns over time. The relative stability of this metric in Brazil suggests that the complexity of inpatient management and discharge practices has remained uniform, despite external stressors such as the pandemic. The modest extension in 2020, coinciding with the pandemic onset, may reflect delayed admissions and more severe presentations due to postponed care. Finally, the inflation-adjusted total cost of R$ 11.38 million (2015-2024) underscores a meaningful economic footprint and disease burden relative to the rarity of PBC. Our results also highlight a pronounced regional gradient, with the Southeast concentrating both the highest absolute economic burden and the highest mean cost per admission, suggestive of greater case complexity and/or concentration of specialized care. Interestingly, mean cost per hospital admission in Brazil (USD 725.10) was comparable to that reported in Italy, where inpatient activities were estimated to cost €473.7 per patient annually^27^.

From a public health perspective, the results emphasize the need to strengthen RWE infrastructures for rare liver diseases in Brazil. The use of administrative datasets, as demonstrated in this study, represents a valuable approach for monitoring disease trends, evaluating healthcare equity, and identifying unmet needs in clinical practice. This investigation has some inherent limitations. A central consideration when interpreting these findings is that the analysis was event-based. As an ecological analysis based on administrative data, it cannot distinguish individual patients, and therefore multiple hospitalizations by the same person may be counted as separate events. Hospitalization rates may overrepresent the burden among patients with recurrent decompensation, older age, limited outpatient access, or repeated transfers between facilities. Second, in-hospital mortality reflects the proportion of admissions ending in death rather than patient-level mortality or case fatality. Consequently, the results should be interpreted as indicators of inpatient healthcare utilization and admission-level outcomes, not as estimates of the incidence, prevalence, or survival of individuals with PBC. Additionally, the use of a constant population denominator from 2025 IBGE estimates, while necessary for internal consistency, may slightly distort annual rate precision. Importantly, using the total Brazilian population as the denominator, rather than the proportion exclusively dependent on the public health system (approximately 76% of the population), may have led to underestimation of the reported rates. The absence of variables on comorbidities, sex distribution, medication use, or liver transplantation restricts clinical granularity. It is also not possible to determine if PBC or liver-related complications were the main reason for each hospital admission. Finally, PBC may be under-ascertained in hospital data, as admissions for cirrhosis decompensation or nonhepatic reasons are often not coded as PBC (ICD-10). Notably, up to 2021 the most frequent liver-related causes of death in Brazil were malignant neoplasm of the liver/intrahepatic bile ducts, alcohol-associated liver disease, and cirrhosis (ICD-10 C22, K70, K74), similar to Europe and the United States^20^ ^,^ ^28^ ^,^ ^29^. Nonetheless, these limitations are intrinsic to population-level RWE studies and do not detract from the observed temporal and regional trends. Finally, given the annual aggregation and limited number of time points, ITS findings should be interpreted as an exploratory segmented descriptive analysis rather than a formal causal evaluation of the COVID-19 pandemic.

## CONCLUSION

In conclusion, hospitalizations for PBC in Brazil remain infrequent but are associated with substantial in-hospital mortality. The COVID-19 pandemic appeared to be associated with a temporary increase in hospitalization rates, followed by normalization in subsequent years. However, these patterns should be interpreted as exploratory and should not be considered definitive evidence of a causal pandemic effect. Despite the stability of hospital stays, the persistent mortality burden highlights a critical need for earlier diagnosis, equitable drug access, and stronger hepatology referral networks. These findings provide essential RWE to inform healthcare planning, clinical guidelines, and resource allocation for autoimmune liver diseases in Latin America.

## Data Availability

data-available-upon-request
